# Almond intake alters the acute plasma dihydroxy-octadecenoic acid (DiHOME) response to eccentric exercise

**DOI:** 10.3389/fnut.2022.1042719

**Published:** 2023-01-09

**Authors:** David C. Nieman, Ashraf M. Omar, Colin D. Kay, Deepak M. Kasote, Camila A. Sakaguchi, Ankhbayar Lkhagva, Mehari Muuz Weldemariam, Qibin Zhang

**Affiliations:** ^1^Human Performance Laboratory, Appalachian State University, North Carolina Research Campus, Kannapolis, NC, United States; ^2^UNCG Center for Translational Biomedical Research, University of North Carolina at Greensboro, North Carolina Research Campus, Kannapolis, NC, United States; ^3^Department of Food, Bioprocessing and Nutrition Sciences, Plants for Human Health Institute, North Carolina State University, North Carolina Research Campus, Kannapolis, NC, United States

**Keywords:** almonds, exercise, oxylipins, inflammation, cytokines, metabolites, (poly)phenols

## Abstract

**Introduction:**

This investigation determined if 4-weeks ingestion of nutrient-dense almonds mitigated post-exercise inflammation and muscle soreness and damage.

**Methods:**

An acute 90-min of eccentric exercise (90-EE) was used to induce muscle damage in 64 non-obese adults not engaging in regular resistance training (ages 30–65 years, BMI < 30 kg/m^2^). Using a parallel group design, participants were randomized to almond (AL) (57 g/d) or cereal bar (CB) (calorie matched) treatment groups for a 4-week period prior to the 90-EE (17 exercises). Blood and 24-h urine samples were collected before and after supplementation, with additional blood samples collected immediately post-90-EE, and then daily during 4 additional days of recovery. Changes in plasma oxylipins, urinary gut-derived phenolics, plasma cytokines, muscle damage biomarkers, mood states, and exercise performance were assessed.

**Results:**

The 90-EE protocol induced significant muscle damage, delayed onset of muscle soreness (DOMS), inflammation, reduced strength and power performance, and mood disturbance. Interaction effects (2 group × 7 time points) supported that AL vs. CB was associated with reduced post-exercise fatigue and tension (*p* = 0.051, 0.033, respectively) and higher levels of leg-back strength (*p* = 0.029). No group differences were found for post-90-EE increases in DOMS and six cytokines. AL was associated with lower levels of serum creatine kinase immediately- and 1-day post-exercise (*p* = 0.034 and 0.013, respectively). The 90-EE bout increased plasma levels immediately post-exercise for 13 oxylipins. Interaction effects revealed significantly higher levels for AL vs. CB for 12,13-DiHOME (*p* < 0.001) and lower levels for 9,10-DiHOME (*p* < 0.001). Urine levels increased in AL vs. CB for seven gut-derived phenolics including 5-(3′,4′-dihydroxyphenyl)-γ-valerolactone that was inversely related to changes in plasma 9,10-DiHOME (*r* = −0.029, *p* = 0.021).

**Discussion:**

These data support some positive effects of almond intake in improving mood state, retaining strength, decreasing muscle damage, increasing the generation of gut-derived phenolic metabolites, and altering the plasma oxylipin DiHOME response to unaccustomed eccentric exercise in untrained adults. The elevated post-exercise plasma levels of 12,13-DiHOME with almond intake support positive metabolic outcomes for adults engaging in unaccustomed eccentric exercise bouts.

## Introduction

Almonds provide a unique and complex nutrient and polyphenol mixture that may support metabolic recovery from stressful levels of exercise. A 57-g serving of almonds has 324 calories with 12 g of protein, 28 g of fat (17 g monounsaturated, 6.6 g polyunsaturated, 2.6 g saturated), 12 g of carbohydrates, and 6.6 g of fiber (1). Almonds are a significant source of vitamin E (96% of the Daily Value, DV, per 57-g serving), magnesium (36% DV), manganese (53% DV), copper (64% DV), the amino acid arginine (1.4 g), and branched chain amino acids (1.75 g).

The total (poly)phenol content of almonds is 164 mg per 57-g serving (1–3). (Poly)phenols in almonds include flavonols (kaempferol, isorhamnetin, and quercetin), flavanols (several types of catechins), flavanones (eriodictyol and naringenin), and simple phenolic acids such as protocatechuic acid (3,4-dihydroxybenzoic acid) and vanillic acid (4-hydroxy-3-methoxybenzoic acid) (2, 4). Proanthocyanidins (PACs) are the most abundant class of (poly)phenols in almonds and occur as procyanidin mixtures of oligomers and polymers consisting of the monomers (+)-catechin and/or (−)-epicatechin. (Poly)phenols from almonds and other plant foods undergo extensive biotransformation in the large intestine where microbial catabolism produces gut-derived phenolic metabolites that exert bioactive effects after reabsorption (5–8).

In clinical trials, almond consumption has been related to reductions in insulin resistance, and serum levels of low-density lipoprotein (LDL), lipoprotein(a), and C-reactive protein (CRP) and other biomarkers of inflammation (9–14). Almond (poly)phenolics possess anti-inflammatory, vasodilatory and antioxidant activities (15–19). Four-weeks intake of almonds has been linked to reduced oxidized LDL and fatty acid peroxidation malondialdehyde (MDA) and creatinine-adjusted urinary isoprostane output (17, 18). Limited data suggest reduced levels of depression with increased almond intake that may in part be due to almond-related anti-inflammatory and antioxidant effects (20). Although unstudied, these data suggest that almond ingestion has the potential to reduce inflammation and oxidative stress, improve mood state, and mitigate muscle soreness and damage within an exercise-stress context.

During the past decade, technological advances have created opportunities for multi-omics and systems biology approaches to be applied to sports nutrition studies. Oxylipins are bioactive, oxidized lipids that are involved in the post-exercise inflammation response. Recent evidence published by our research group has shown that exercise-induced increases in plasma oxylipin levels can be altered through nutritional interventions including added intake of (poly)phenols (21, 22). Stressful levels of exercise increase plasma levels of oxylipins, with most of these generated from the metabolism of n-6 and n-3 polyunsaturated fatty acids (PUFAs) by cyclooxygenase (COX), lipoxygenase (LOX), and cytochrome P450 (CYP) enzyme systems (21–23). Limited evidence indicates that polyphenols may regulate these enzymes and alter the production of proinflammatory oxylipins (24–26). Increased intake of PUFAs can also alter plasma levels of oxylipins (27–30).

This randomized clinical trial determined if 4-weeks of almond vs. cereal bar supplementation would improve inflammation resolution and metabolic recovery from a 90-min bout of eccentric exercise in adults unaccustomed to resistance training. Emphasis was placed on the measurements of inflammatory oxylipins and cytokines that regulate the post-exercise inflammatory process. Almond (poly)phenol metabolites in 24-h urine samples were measured and related to inflammation-based outcomes to determine potential underlying mechanisms. Other outcomes included exercise performance, muscle soreness and damage, and mood states.

## Materials and methods

### Study participants

**FIGURE 1:**
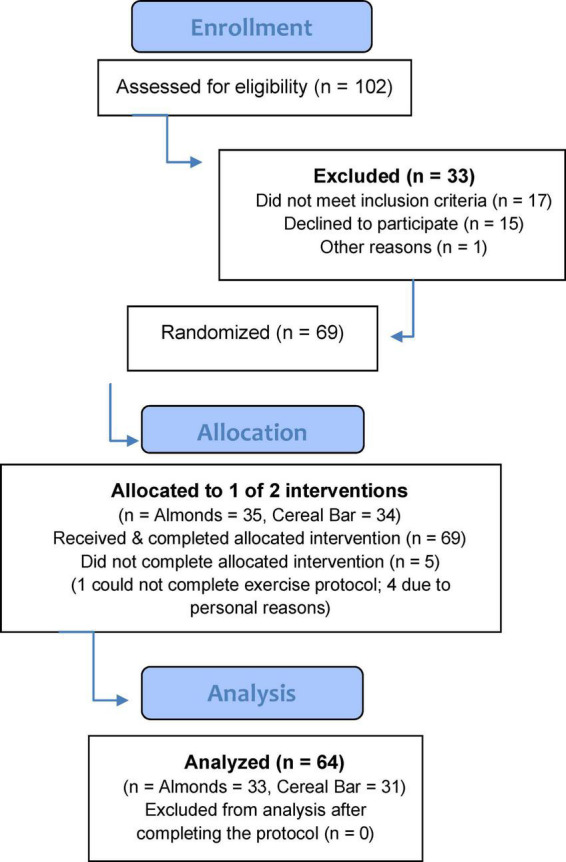
CONSORT subject flow diagram.

Healthy, non-smoking male and female study participants were invited to take part in this study if they met the inclusion criteria including 30–65 years of age, body mass index (BMI) less than 30 kg/m^2^, not engaged in regular resistance training (less than 3 sessions per week), and a willingness to avoid the use of protein supplements, large-dose vitamin/mineral and herbal supplements, and anti-inflammatory medications during the project. After 102 participants were assessed for eligibility, 69 were randomized to almond (AL) or cereal bar (CB) groups, with 64 completing the protocol (Figure 1). The study participant number (at least 30 per group) provided more than 84% power to detect a difference with an effect size 0.7 at alpha 0.05 using two-sided *t*-tests. Participants voluntarily signed the informed consent, and procedures were approved by the university’s Institutional Review Board. Trial Registration: ClinicalTrials.gov, U.S. National Institutes of Health, identifier: NCT04958018.

### Study design

This study employed a randomized, parallel group design with a 4-week supplementation period, 90-min eccentric exercise bout challenge, and seven lab visits at the Appalachian State University Human Performance Laboratory (HPL) at the North Carolina Research Campus, Kannapolis, NC. The parallel group design was utilized similar to a previous study in our lab because of the “repeat exercise bout” effect (i.e., post-exercise muscle damage and inflammation are reduced when the same bout is repeated within days and weeks of the first bout) (31).

The 4-week supplementation period was based on previous studies with almonds showing bioactive effects within this length of time (1). Participants in the almond group consumed 57 g almonds each day in split doses (half in the morning, half in the afternoon). Participants in the snack group ingested a calorie-matched snack bar in split doses (Nutri-Grain cereal bars, apple-cinnamon, 324 calories/day, 60 g carbohydrate, 8.8 g fat, 5 g protein, 25% DV each for iron, vitamin A, riboflavin, vitamin B6, calcium, thiamin, niacin, and zinc) (Kellogg’s, Battle Creek, MI). Table 1 summarizes key nutrient differences between 324 calorie portions of almonds and cereal bars.

**TABLE 1:** Nutrient comparison between one serving of raw almonds (57 g) and a calorie-matched amount of cereal bars (93 g).

Key nutrients	57 g raw almonds	93 g cereal bars	Male RDA	Female RDA
Kilocalories	324	324		
Protein (g)	11.9	5		
Arginine (g)	1.4			
Total fat (g)	28.0	8.8		
Monounsaturated fat (g)	17.7			
Carbohydrate (g)	12.1	60		
Fiber (g)	7.0	2.5		
Sugars (g)	2.44	32.5		
Calcium (mg)	151	32.5	1,000	1,000
Iron (mg)	2.1	4.5	8	18
Magnesium (mg)	151		400	320
Zinc (mg)	1.76	2.75	11	8
Copper (mg)	0.58		0.9	0.9
Manganese (mg)	1.23		2.3	1.8
Vitamin E (μg alpha-TE)	14.4		15	15

Seven blood samples were collected before and after 4-weeks supplementation (overnight fasted state), immediately post-exercise (within 5 min after exercise ended), and then each morning (overnight fasted state) during 4 days of recovery. Two 24-h urine samples were collected (total urine output) before and after the 4-week supplementation period. Ascorbic acid (300 mg) and boric acid (10 g) were added to the 24-h urine containers to help stabilize the phenolic molecules. After each of the blood draws (each overnight fasted except immediately post-exercise), participants completed the abbreviated Profile of Mood States (POMS) questionnaire using procedures described in an earlier study in our lab (32), provided a muscle soreness rating using a 1–10 scale (DOMS) (33), and completed the muscle function testing protocol. Participants recorded all food and beverage intake for 3 days at the end of the 4-week supplementation period to assess the background diet. Macro- and micro-nutrient, and flavonoid intake was assessed using the Food Processor dietary analysis software system with an adapted nutrient database as described previously (34) (Version 11.1, ESHA Research, Salem, OR, USA).

Study participants signed the consent form and were given a complete orientation to the study protocol during the first lab visit. The orientation included an introduction with practice of the exercises included in the eccentric exercise bout and the muscle function tests. During the second lab visit (overnight fasted, pre-supplementation), study participants provided a blood sample, turned in the 24-h urine collection bottle, and provided responses to POMS and DOMS questionnaires. Height and body weight were assessed, with body composition measured using the BodPod system (Cosmed, Rome, Italy). Participants then performed five muscle function tests as described previously (31): vertical jump, bench press, leg-back strength, 60-yard shuttle run test, and anaerobic power through the 30-s Wingate test. Briefly, in the vertical jump test, participants jumped as high as possible with one hand, and tapped the measuring device (Vertec vertical jump apparatus, Questtek Corp., Northridge, CA, USA). In the bench press to exhaustion, participants laid down supine on the bench, and bench pressed a weighted bar equal to 50% (females) or 75% (males) of their body weight as many times as possible at a rate of 30 lifts per minute. Leg/lower back isometric lifting strength was assessed with a dynamometer (Lafayette Instruments, Lafayette, IN, USA). The Lode cycle ergometer (Lode B.V., Groningen, Netherlands) was used for the 30-s Wingate cycling test. The workload was adjusted to the body mass of the subject (0.7 Newton meters per kilogram), and participants cycled at maximal speed for 30 s. The peak and total wattage power output were recorded and adjusted to body mass. In the 60-yard shuttle run test, participants sprinted progressing distances back and forth (5, 10, and 15 yards), with the total time recorded in seconds. Study participants were then provided a 4-weeks supply of almonds or cereal bars and told to return in 4 weeks for the eccentric muscle exercise bout. Supplementation compliance was monitored with regular email messages and the return of supplement wrappers.

Participants returned for their third lab visit after the 4-week supplementation period in an overnight fasted, turned in 3-day food records and 24-h urine bottles, gave responses to DOMS and POMS questionnaires, and provided a blood sample. Almond and cereal bar supplements (half doses) were ingested followed by muscle function testing. As described previously, participants then engaged in a 90-min eccentric exercise bout that consisted of 17 different exercises, most with 2–3 sets and 30–60 s of rest between sets (31). Participants returned at 7:00 a.m. in an overnight fasted state 4 days in a row after the eccentric exercise bout, and provided blood samples, POMS and DOMS ratings, and repeated the five physical fitness tests. Almond and cereal bar supplementation was continued during this 4-day recovery period.

### Sample analysis

Blood samples were collected in serum separation tubes (SST) and ethylenediaminetetraacetic acid (EDTA) containing blood collection tubes. SST were spun at 2,300 rpm for 15 min after being allowed to clot for 15 min. Serum creatine kinase and myoglobin were analyzed each day samples were collected using Labcorp services (Burlington, NC). Plasma aliquots were prepared from ethylenediaminetetraacetic acid (EDTA) containing blood collection tubes and stored in a −80°C freezer until analysis for cytokines and oxylipins after the study was completed.

#### Plasma cytokines

Plasma IL-6, IL-8, IL-10, and monocyte chemotactic protein (MCP-1) were measured with the multiplexed immunoassay platform –Ella™ (Protein Simple, CA) (34). Briefly, each individual sample was diluted twofold and 50 μl of the diluted sample was loaded to each well on the 32-sample cartridge or 72-sample cartridge, and the concentration of each cytokine was determined with built-in calibration curves. For quality control purposes and measurement reproducibility, aliquots of pooled plasma samples were processed the same as each individual sample to control the variation between cartridges.

#### Plasma oxylipins

Plasma arachidonic acid (ARA), eicosapentaenoic acid (EPA), docosahexaenoic acid (DHA), and oxylipins were analyzed using a liquid chromatography-multiple reaction monitoring mass spectrometry (LC-MRM-MS) method as fully described elsewhere (35). Resultant data files were processed with Skyline (36), and the auto-integrated peaks were inspected manually. Concentrations of each oxylipin were determined from calibration curves of each analyte, which were constructed by normalizing to the selected deuterated internal standards followed by linear regression with 1/x weighting (Data Sheet 2). The coefficient of variation for the quality control standards was <15% as reported in the method development paper (35).

#### Urine phenolic metabolite analysis

A targeted UPLC-MS/MS method was developed to detect 105 almond analytes/metabolites and quantitated against 70 commercially available authentic reference standards. The remaining 35 phenolic metabolites (including Phase II conjugates) having no available reference standards were annotated based on fragmentation pattern, and putatively quantified using surrogate reference standard curves derived from their closest structural reference standard (Supplementary Data Sheet 1). Human urine metabolites (50 μl samples) were isolated using 96-well solid phase extraction (SPE; Strata™-X Polymeric Reversed Phase, microelution 2 mg/well) following previously published methods (22). Metabolite separation and quantitative analysis was performed on a Exion ultra-high performance liquid chromatography system coupled with a triple quadrupole mass spectrometer (SCIEX, QTRAP 6500+, Framingham, MA, USA) following previously published methodology, with slight modification (22). Briefly, quantitation was performed using SCIEX OS (v.2.0.0.45330, SCIEX) software. Internal standards were L-tyrosine-13C9,15N, resveratrol-13C6, hippuric acid 13C, 13C6 4-hydroxybenzoic acid propyl ester, and phlorizin dehydrate (Sigma-Aldrich). Calibration curves consisting of 11 concentrations (1 nM–10 μM) were established for 70 analytes by spiking reference standards in synthetic human urine (Surine™ S-020, Sigma-Aldrich Corporation, St. Louis, MO, USA).

### Statistical analysis

The data are expressed as mean ± SE and were analyzed using the generalized linear model (GLM), repeated measures ANOVA module in SPSS (IBM SPSS Statistics, Version 28.0, IBM Corp., Armonk, NY, USA). The statistical model utilized the between-subjects approach: 2 (groups) × 7 (time points) repeated measures ANOVA and provided time (i.e., the collective effect of the eccentric exercise bout) and interaction effects (i.e., whether the data pattern over time differed between groups). If the interaction effect was significant (*p* ≤ 0.05), then *post hoc* analyses were conducted using Student’s *t*-tests comparing time point contrasts between groups. An alpha level of *p* ≤ 0.008 was used after Bonferroni correction for 6 multiple tests, with *p*-values ≤ 0.05–0.008 regarded as trends. The positive false discovery rate (FDR or “*q*-value”) was calculated for multiple testing correction of the plasma oxylipin and urine metabolite data. Statistical significance for the interaction effects were set at *q* ≤ 0.10 and *q* ≤ 0.20, respectively, due to the large number of these variables. Pearson correlation coefficients and the corresponding *p*-values were calculated between targeted oxylipin and urine metabolites. Between group study participant characteristics were contrasted using independent *t*-tests.

## Results

Of 102 adults assessed for eligibility, 69 were randomized to almond (AL) and cereal bar (CB) groups (Figure 1). Characteristics for the *n* = 64 study participants completing all aspects of the study protocol are summarized in Table 2. The proportion of males and females between groups did not differ (chi-square statistic = 0.0428, *p*-value = 0.836). The almond and cereal bar groups did not differ in age, weight, height, and body composition (body fat %). Male and female study participants did not differ significantly in key outcomes of this study and were thus combined in the group comparisons.

**TABLE 2:** Subject characteristics for the almond (*n* = 33, 20 males, 13 females) and cereal bar (*n* = 31, 18 males, 13 females) groups.

	Supplement group	Mean	*t*-test *P*-value
Age (year)	Almond	46.1 ± 1.5	0.744
	Cereal bar	46.8 ± 1.6	
Weight (kg)	Almond	77.4 ± 2.0	0.387
	Cereal bar	79.7 ± 1.8	
Height (cm)	Almond	173 ± 1.7	0.338
	Cereal bar	172 ± 1.4	
Body fat (%)	Almond	27.4 ± 1.4	0.533
	Cereal bar	28.8 ± 1.8	

Three-day food records collected at the end of the 4-week supplementation period to assess the background diet (not including the supplements) revealed no significant differences in energy, carbohydrate, and micronutrient intake between groups (data not shown). For the entire group, energy intake of the background diet averaged 1,730 ± 79 kcal/day (7.24 ± 0.33 MJ/day), with carbohydrate, protein, fat, and alcohol representing 43.6 ± 1.3%, 18.8 ± 0.9%, 35.1 ± 0.9%, and 3.6 ± 0.7%, respectively of total energy. Total flavonoid intake averaged 131 ± 35.4 mg/day. The almond and cereal bar supplements added 324 kcal/day to energy intake.

Performance data for each group across seven time points are summarized in Table 3. The 90-min eccentric exercise bout influenced shuttle run time, leg/back strength, bench press repetitions, and 30-s Wingate peak and mean power (all time effects, *p* ≤ 0.004). The patterns of change over time (interaction effects) between groups for the performance tests were not different except for leg/back strength (*p* = 0.029), with higher levels in AL compared to CB during recovery. No recovery time point was significantly different between groups after Bonferroni correction.

**TABLE 3:** Performance data before and after 4-weeks supplementation, and immediately post 90 min eccentric exercise and in an overnight fasted state during 4 days of recovery (AL = almond, *n* = 33; CB = cereal bar, *n* = 31) (means ± SE).

Variable	Group	Pre-study	4-weeks post-suppl	0 h post-ex	1 day post-ex	2 days post-ex	3 days post-ex	4 days post-ex	Time; interaction *P*-values
Vertical jump (cm)	AL	40.4 ± 2.16	40.6 ± 2.11	41.4 ± 2.21	40.4 ± 2.18	39.9 ± 2.11	40.4 ± 2.24	41.4 ± 2.18	0.002, 0.083
	CB	38.4 ± 1.91	40.1 ± 1.91	39.9 ± 2.24	40.0 ± 2.16	39.9 ± 2.11	41.1 ± 2.13	41.4 ± 2.08	
Shuttle run (seconds)	AL	18.0 ± 0.48	18.2 ± 0.51	20.2 ± 1.17	18.9 ± 0.62	19.1 ± 0.66	18.4 ± 0.53	18.5 ± 0.56	<0.001, 0.857
	CB	18.9 ± 0.59	19.0 ± 0.59	21.7 ± 1.11	20.3 ± 0.75	20.4 ± 0.84	19.5 ± 0.64	19.7 ± 0.62	
Leg/back strength (kg/kg body mass)	AL	1.65 ± 0.10	1.66 ± 0.10	1.60 ± 0.10	1.64 ± 0.10	1.66 ± 0.09	1.68 ± 0.10	1.70 ± 0.11	0.004, 0.029
	CB	1.53 ± 0.10	1.47 ± 0.09	1.40 ± 0.10	1.38 ± 0.10	1.51 ± 0.10	1.46 ± 0.09	1.45 ± 0.09	
Bench press (reps)	AL	12.0 ± 1.10	12.1 ± 1.04	9.18 ± 1.09	11.2 ± 1.12	12.1 ± 1.08	12.9 ± 1.11	13.5 ± 1.05	<0.001, 0.338
	CB	11.4 ± 0.9	12.4 ± 1.07	8.10 ± 1.24	10.9 ± 1.30	11.4 ± 1.15	12.6 ± 1.09	14.1 ± 1.09	
Wingate peak power (watts/kg)	AL	6.52 ± 0.33	6.51 ± 0.36	5.66 ± 0.33	6.23 ± 0.35	6.35 ± 0.33	6.11 ± 0.32	6.33 ± 0.33	<0.001, 0.316
	CB	6.30 ± 0.41	5.95 ± 0.36	5.22 ± 0.37	5.59 ± 0.38	5.81 ± 0.40	5.73 ± 0.41	5.79 ± 0.37	
Wingate mean power (watts/kg)	AL	5.12 ± 0.30	5.03 ± 0.29	4.34 ± 0.29	4.82 ± 0.28	4.92 ± 0.28	4.80 ± 0.30	4.99 ± 0.29	<0.001, 0.372
	CB	4.90 ± 0.34	4.63 ± 0.32	4.02 ± 0.34	4.36 ± 0.33	4.58 ± 0.35	4.55 ± 0.34	4.64 ± 0.32	

Suppl, supplementation; Post-ex, post-90 min eccentric exercise bout.

Muscle soreness (DOMS), damage biomarkers (creatine kinase, myoglobin), and POMS data are summarized in Table 4. The 90-min eccentric exercise bout increased muscle soreness and damage, with an increase in total mood disturbance POMS scores (all time effects, *p* ≤ 0.006). The patterns of change over time for muscle soreness (DOMS) (*p* = 0.187) and total mood disturbance POMS scores (*p* = 0.130) did not differ between groups. The patterns of change for two POMS domains were different between groups with lower scores in AL compared to CB during recovery for fatigue (*p* = 0.051) and tension (*p* = 0.033). Interaction statistics were non-significant for serum creatine kinase and serum myoglobin when considering all seven time points (Table 4). The pattern of change over three time points (pre-exercise, immediately post-exercise, 1 day-post-exercise) tended to be different between groups (*p* = 0.066) for creatine kinase, with lower values in the almond group for the two recovery time points (*p* = 0.034 and *p* = 0.013, respectively). The pattern of change for serum glucose did not differ between groups (*p* = 0.769, data not shown). Serum glucose concentrations immediately post-exercise did not differ between AL and CB groups (5.31 ± 0.13 and 5.31 ± 0.23 mmol/L, respectively), and were similar to overnight fasted, pre-exercise values (5.09 ± 0.25 and 5.32 ± 0.08 mmol/L, respectively).

**TABLE 4:** Delayed onset of muscle soreness (DOMS), and muscle damage data before and after 4-weeks supplementation, and immediately post 90 min eccentric exercise and in an overnight fasted state during 4 days of recovery (AL = almond, *n* = 33; CB = cereal bar, *n* = 31) (means ± SE).

Variable	Group	Pre-suppl	4-weeks post-suppl	0 h post-ex	1 day post-ex	2 days post-ex	3 days post-ex	4 days post-ex	Time; interaction *P*-values
DOMS (1–10 scale)	AL	1.83 ± 0.21	1.73 ± 0.17	3.80 ± 0.32	6.24 ± 0.31	5.85 ± 0.32	3.97 ± 0.28	2.59 ± 0.23	<0.001, 0.187
	CB	1.73 ± 0.21	1.90 ± 0.20	4.56 ± 0.41	5.98 ± 0.30	5.98 ± 0.39	4.37 ± 0.40	3.18 ± 0.35	
POMS total mood disturbance	AL	93.5 ± 1.7	90.0 ± 3.2	98.1 ± 1.8	95.8 ± 1.6	94.9 ± 1.6	94.3 ± 1.3	91.4 ± 1.3	0.007, 0.130
	CB	95.9 ± 3.0	97.2 ± 3.2	103 ± 2.1	102 ± 2.5	101 ± 2.7	97.6 ± 2.7	97.8 ± 3.0	
POMS fatigue	AL	1.79 ± 0.45	2.27 ± 0.68	7.18 ± 0.86	3.79 ± 0.71	3.42 ± 0.64	3.15 ± 0.56	1.79 ± 0.38	<0.001, 0.051
	CB	2.23 ± 0.80	1.97 ± 0.62	8.55 ± 0.96	5.87 ± 0.88	4.90 ± 0.87	3.48 ± 0.73	3.68 ± 0.82	
POMS tension	AL	1.52 ± 0.40	1.06 ± 0.26	1.15 ± 0.34	0.52 ± 0.17	0.45 ± 0.21	0.52 ± 0.19	0.24 ± 0.18	0.006, 0.033
	CB	1.55 ± 0.59	2.97 ± 0.80	0.87 ± 0.30	1.35 ± 0.43	1.58 ± 0.47	1.16 ± 0.48	1.19 ± 0.51	
Creatine kinase (Units/L)	AL	174 ± 23.5	165 ± 24.3	210 ± 26.5	403 ± 40.0	550 ± 232	758 ± 333	841 ± 350	<0.001, 0.151
	CB	162 ± 29.2	185 ± 35.5	317 ± 64.4	728 ± 153	545 ± 130	481 ± 120	513 ± 136	
Serum myoglobin (ng/ml)	AL	35.1 ± 2.7	32.8 ± 2.2	147 ± 13.5	66.1 ± 8.2	108 ± 53.6	107 ± 38.8	83.5 ± 21.1	<0.001, 0.299
	CB	32.9 ± 2.8	36.1 ± 3.2	202 ± 25.5	86.7 ± 15.8	56.1 ± 9.8	79.7 ± 19.9	70.6 ± 12.6	

Suppl, supplementation; Post-ex, post-90 min eccentric exercise bout.

Plasma cytokine data are summarized in Table 5. Transient and modest increases in plasma levels for IL-6, IL-8, IL-10, and MCP-1 were measured in response to the 90-min eccentric exercise bout (time effects, all *p*-values ≤ 0.008). The pattern of changes in plasma levels for these four cytokines did not differ significantly between groups.

**TABLE 5:** Plasma cytokine data before and after 4-weeks supplementation, and immediately post 90 min eccentric exercise and in an overnight fasted state during 4 days of recovery (AL = almond, *n* = 33; CB = cereal bar, *n* = 31) (means ± SE).

Variable	Group	Pre-suppl	4-weeks post-suppl	0 h post-ex	1 day post-ex	2 days post-ex	3 days post-ex	4 days post-ex	Time; interaction *P*-values
IL-6	AL	1.39 ± 0.16	1.33 ± 0.19	2.63 ± 0.38	1.43 ± 0.15	1.23 ± 0.12	1.43 ± 0.29	1.74 ± 0.25	<0.001, 0.323
	CB	1.61 ± 0.17	1.59 ± 0.21	2.83 ± 0.35	1.78 ± 0.32	1.42 ± 0.15	1.46 ± 0.17	1.25 ± 0.10	
IL-8	AL	3.74 ± 0.55	3.56 ± 0.40	3.92 ± 0.27	3.70 ± 0.37	3.38 ± 0.32	3.31 ± 0.28	3.09 ± 0.28	0.001, 0.828
	CB	3.51 ± 0.37	3.77 ± 0.50	4.18 ± 0.39	3.88 ± 0.50	3.77 ± 0.44	3.70 ± 0.46	3.61 ± 0.33	
IL-10	AL	2.13 ± 0.20	1.93 ± 0.12	2.91 ± 0.45	1.95 ± 0.09	1.87 ± 0.12	1.96 ± 0.12	1.92 ± 0.16	0.008, 0.251
	CB	1.84 ± 0.10	1.77 ± 0.13	2.48 ± 0.26	2.00 ± 0.15	1.78 ± 0.13	1.69 ± 0.11	1.89 ± 0.11	
MCP-1	AL	138 ± 6.0	130 ± 5.9	151 ± 8.5	150 ± 8.5	127 ± 5.0	131 ± 4.8	124 ± 7.5	<0.001, 0.107
	CB	137 ± 7.7	138 ± 8.2	156 ± 8.3	140 ± 7.3	134 ± 7.1	120 ± 5.1	129 ± 5.5	

Suppl, supplementation; Post-ex, post-90 min eccentric exercise bout.

**FIGURE 2:**
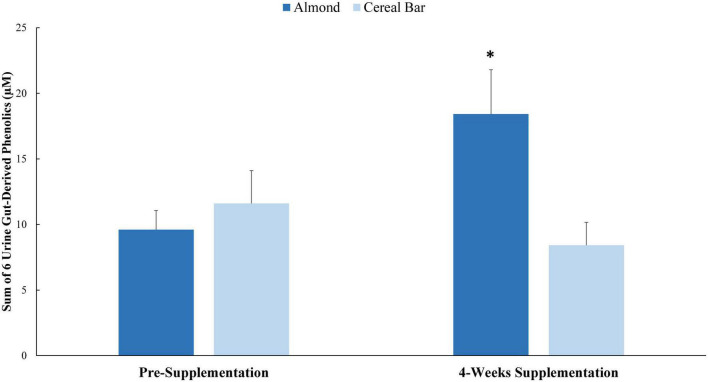
Pre-study and 4-week supplementation data for the composite variable of six urine gut-derived phenolics (interaction effect *p*-value = 0.0013; *q*-value = 0.1258). *Group contrast of the increase relative to pre-supplementation, *P* = 0.0013. Sum of: 5-(3′,4′-Dihydroxyphenyl)- γ-valerolactone; hydroxybenzoic acid-sulfate* (2 isomers of unknown structural configuration); 3-(3-hydroxyphenyl)propanoic acid-5-sulfate; benzoic acid-4-sulfate; 3-(3-hydroxyphenyl)propanoic acid-5-O-glucuronide; 3-(4-methoxyphenyl)propanoic acid-sulfate.

Pre- and post-4 weeks supplementation 24 h urine volumes for the AL (1,907 ± 173, 1,870 ± 148 ml) and CB (1,963 ± 133, 1,788 ± 134 ml) groups were not significantly different between groups (interaction *p*-value = 0.419). Of 105 targeted phenolic metabolites detected in the urine samples (Data Sheet 1), seven had interaction effects with *p*-values ≤ 0.05 (Table 6). Six of these (minus hippuric acid) were combined into a composite variable (Figure 2). Pre-study and 4-week supplementation data for the composite variable indicated a significant increase in the AL compared to CB group (interaction effect *p*-value = 0.0013, *q*-value = 0.1258).

**TABLE 6:** Urine concentrations (24 h collection) of gut-derived phenolics before and after 4-weeks supplementation with almonds (57 g/d) or cereal bars (calorie matched).

			Post-4-weeks			
	Pre-supplementation		supplementation		Interaction effect	
Variable (μM)	Almond	Snack bar	Almond	Snack bar	*P*-value	FDR *q*-value
Hippuric acid	39.0 ± 3.82	44.8 ± 3.12	51.5 ± 3.93	40.0 ± 3.82	0.014	0.458
5-(3′,4′-Dihydroxyphenyl)-γ-valerolactone	4.89 ± 1.59	5.77 ± 1.67	9.11 ± 1.13	3.97 ± 1.15	0.003	0.271
3-(4-Methoxyphenyl) propanoic acid-sulfate	4.13 ± 1.22	5.18 ± 1.29	8.44 ± 0.83	3.96 ± 0.86	0.044	0.590
Benzoic acid-4-sulfate	0.382 ± 0.052	0.348 ± 0.055	0.527 ± 0.044	0.301 ± 0.046	0.036	0.683
Hydroxybenzoic acid-sulfate*	0.078 ± 0.019	0.089 ± 0.020	0.123 ± 0.006	0.058 ± 0.006	0.010	0.466
3-(3-Hydroxyphenyl) propanoic acid-5-sulfate	0.051 ± 0.025	0.083 ± 0.026	0.107 ± 0.009	0.038 ± 0.009	0.027	0.630
3-(3-Hydroxyphenyl) propanoic acid-5-O-glucuronide	0.078 ± 0.029	0.129 ± 0.030	0.119 ± 0.016	0.092 ± 0.016	0.041	0.656

*Sum of 2 isomers of unknown structural configuration.

**FIGURE 3:**
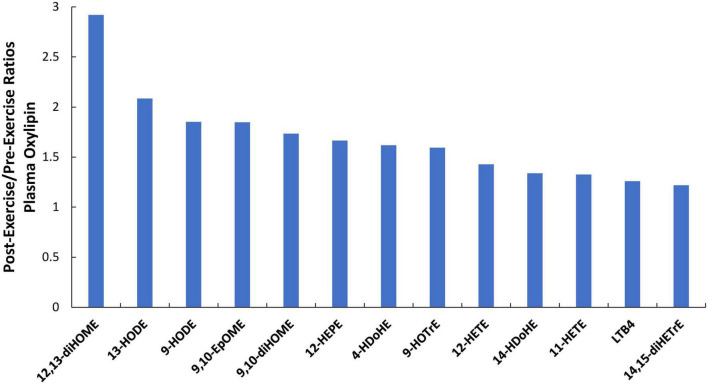
Immediate-post-exercise increases (post-/pre-exercise ratios) in 13 plasma oxylipins for all subjects combined in response to the 90-min eccentric exercise bout. All *q*-values <0.10 after correction for multiple paired *t*-tests.

**FIGURE 4:**
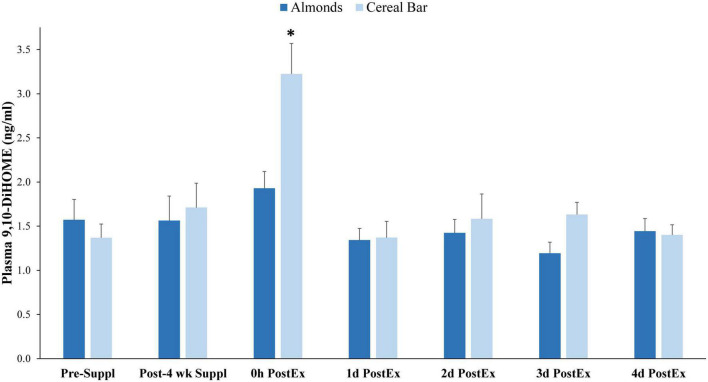
Plasma 9,10-DiHOME data before and after 4-weeks supplementation, and immediately post 90 min eccentric exercise and in an overnight fasted state during 4 days of recovery (AL = almond, *n* = 33; CB = cereal bar, *n* = 31) (means ± SE). Time effect, *p* < 0.001, interaction effect, *p* < 0.001, *q* < 0.10. *p-value = 0.0013, group contrast of the increase relative to pre-supplementation. Suppl, supplementation; PostEx, post-90 min eccentric exercise bout.

**FIGURE 5:**
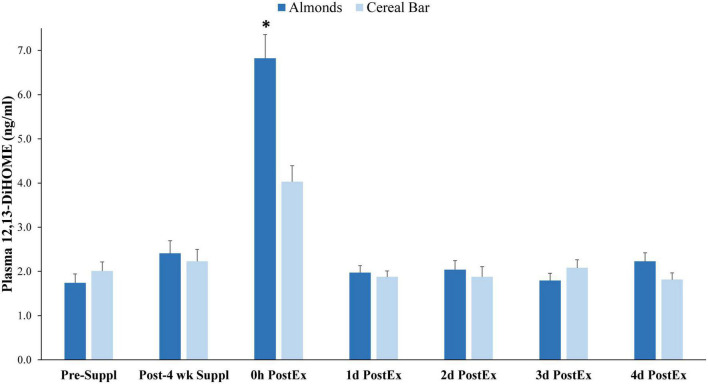
Plasma 12,13-DiHOME data before and after 4-weeks supplementation, and immediately post 90 min eccentric exercise and in an overnight fasted state during 4 days of recovery (AL = almond, *n* = 33; CB = cereal bar, *n* = 31) (means ± SE). Time effect, *p* < 0.001, interaction effect, *p* < 0.001, *q* < 0.10. *p = 0.000013, group contrast of the increase relative to pre-supplementation. Suppl, supplementation; PostEx, post-90 min eccentric exercise bout.

Of 73 oxylipins detected in the study plasma samples (Data Sheet 2), 13 had significant increases immediately post-exercise when compared to pre-supplementation levels (all subjects combined, *q*-values < 0.10) (Figure 3). Of these 13 oxylipins, two had significant interaction effects (*p* ≤ 0.001, *q* < 0.10) (Figures 4, 5). The pattern of change in plasma 9,10-DiHOME differed significantly between groups with the post-exercise increase higher in CB compared to AL (group time point contrast, *p* = 0.0013) (Figure 4). The pattern of change in plasma 12,13-DiHOME also differed between groups, but in contrast, the post-exercise increase was higher in AL compared to CB (*p* = 0.000013) (Figure 5).

**FIGURE 6:**
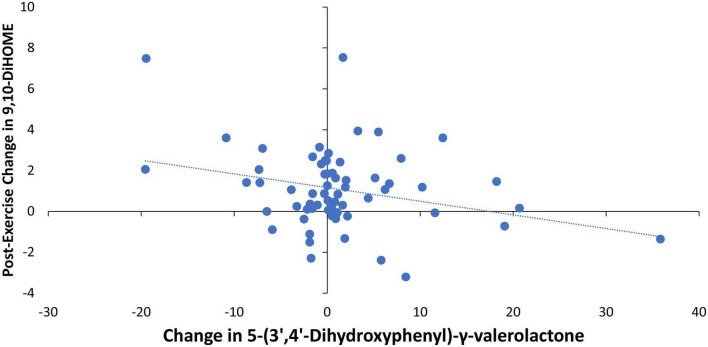
Pearson correlation scatterplot between the change in urine 5-(3′,4′-dihydroxyphenyl)-γ-valerolactone (pre- and post-4-weeks supplementation with almonds or cereal bars) and the post-exercise change in plasma 9,10-DiHOME (*r* = –0.288, *p* = 0.021).

Pearson correlations were calculated for the relationships between 4-weeks changes in the urine gut-derived phenolics listed in Table 5 and the two DiHOMEs (Figures 4, 5). The strongest relationship was between the change in urine 5-(3′,4′-dihydroxyphenyl)-γ-valerolactone and the post-exercise change in plasma 9,10-DiHOME (*r* = −0.288, *p* = 0.021) (Figure 6). The 4-week change in the composite variable of the six urine phenolic metabolites (minus hippuric acid) was not as strongly related to the exercise-induced change in plasma 9,10-DiHOME (*r* = −0.215, *p* = 0.088). No significant relationships were found between changes in urine gut-derived phenolics and 12,13-DiHOME.

## Discussion

The 90-min eccentric exercise protocol induced significant muscle damage, delayed onset of muscle soreness, transient inflammation as measured by increases in plasma cytokines and oxylipins, reduced strength and power performance, and mood disturbance. Some positive effects of almond compared to cereal bar intake emerged in improving mood state (lower fatigue and tension), retaining leg-back isometric strength, and decreasing muscle damage during the first day of recovery. The decrease in post-exercise muscle damage is consistent with data from a previous study in our lab supporting the role of protein and BCAA supplementation in mitigating muscle damage from a 90-min eccentric exercise bout (31). The modest effect of almond intake on mood state within an exercise context is a novel finding but consistent with previously reported linkages between nut intake and mood state (20).

Almond supplementation for 4 weeks increased urine concentrations for seven gut-derived phenolics. Of the 13 oxylipins that increased immediately post-exercise (22–192%), almond compared to cereal bar supplementation had a strong effect in elevating plasma levels of 12,13-DiHOME and lowering 9,10-DiHOME. Despite substantial muscle damage and soreness, the magnitude of increase in plasma cytokine and oxylipin levels was modest compared to those measured after prolonged and intensive cycling or running (21–23). In a previous study, 64 of 67 plasma oxylipins increased in athletes cycling 75 km, and post-/pre-exercise ratios indicated substantial increases (average of 11.8 compared to just 1.7 in the current study) (22). Nearly half of the plasma oxylipins elevated after 75-m cycling came from arachidonic acid, with many generated from the CYP enzyme system with pro-inflammatory effects. The plasma cytokine and oxylipin response to the 90-min eccentric muscle exercise bout was transient and did not parallel the delayed DOMS and muscle damage response during the 4-day recovery period. Markworth et al. (37, 38) have also reported modest, transient increases in both plasma and muscle oxylipin levels following acute resistance exercise and concluded that oxylipins were more involved with regulating the onset and resolution of acute exercise-induced skeletal muscle inflammation than in longer-term recovery processes.

The influence of exercise and diet on oxylipins is an emerging science and much remains to be explored. We previously showed that acute carbohydrate supplementation (0.8 g/kg each hour of intensive cycling exercise) and 2-weeks blueberry supplementation attenuated post-75-km cycling increases in plasma oxylipins, especially proinflammatory lipid mediators generated from arachidonic acid (ARA)-CYP (21, 22). Study participants ingested half doses of CB with 30 g carbohydrate and AL with 6 g carbohydrate just before engaging in the 90-min eccentric exercise bout. No carbohydrate was consumed during exercise. Pre- and post-exercise serum glucose levels in the current study were similar, and the relatively small amount of carbohydrate ingested before the 90-min eccentric exercise bout is unlikely to have influenced oxylipin generation to a significant degree. The almond supplementation effect on post-exercise plasma DiHOME levels is a novel finding. DiHOMES are generated from CYP-formed epoxy fatty acid metabolites of linoleic acid (EpOMEs) that are rapidly hydrolyzed by the soluble epoxide hydrolase enzyme (sEH) (39). DiHOMEs are relatively abundant in plasma compared to other oxylipins and demonstrate a wide variety of biological effects, having both deleterious and beneficial outcomes depending on tissue concentrations and disease conditions (40).

Post-exercise plasma levels of 12,13-DiHOME were 69% higher in the almond compared to cereal bar group. A previous study showed that aerobic exercise (40–45 min of cycling, 70–75% heart rate reserve) transiently increases plasma levels of 12,13-DiHOME but not 9,10-DiHOME in human subjects (41). Resting 12,13-DiHOME levels (but not 9,10-DiHOME) were positively related to VO2 peak and negative correlated with fat mass and BMI. Using a mouse model, brown adipose tissue (BAT) was determined to be the source of 12,13-DiHOME generation during exercise with positive effects on skeletal muscle fatty acid uptake and mitochondrial respiration. BAT-derived 12,13-DiHOME in another study was shown to improve cardiomyocyte contraction, relaxation, and mitochondrial respiration (42). Thus, 12,13-DiHOME is now viewed as a lipokine that can be elevated with exercise exerting a positive influence on metabolic health and energy regulation (43–47).

In contrast with 12,13-DiHOME, almond compared to cereal bar ingestion was associated with 40% lower post-exercise plasma levels of 9,10-DiHOME. In a previous study conducted by our research group, we showed that pistachio supplementation was associated with a marked elevation in plasma 9,10-DiHOME but not 12,13-DiHOME after 75 km cycling, and this was related to the high levels of raffinose in pistachios and their translocation from the colon to the circulation during exercise (48). Raffinose is at very low levels in almonds, and the lower post-exercise plasma 9,10-DiHOME levels in the almond supplementation group could be due in part to the influence of the nutrient mixture in almonds on CYP and sEH biosynthetic pathways. Of the various nutrients in a 57-g serving of almonds, the antioxidant vitamin E, specifically α-tocopherol, is present in the largest quantity when expressed relative to DV standards. However, data on the influence of vitamin E on CYP or sEH activity is lacking. Linoleic acid (LA, 18:2) is the primary PUFA in almonds (7 g/57 g almond serving), and this fatty acid is a precursor to both LA-CYP generated DiHOMES. However, the increased intake of 7 g LA/d in the almond group does not explain the very specific and contrasting changes in post-exercise plasma levels of 9,10-DiHOME and 12,13-DiHOME.

In this study, 4-weeks of almond intake increased urine levels of seven gut-derived phenolics, and a modest inverse relationship was found between 5-(3′,4′-dihydroxyphenyl)-γ-valerolactone (3,4-diHPV) and plasma 9,10-DiHOME but not 12,13-DiHOME. This implies a selective effect of 3,4-diHPV on sEH activation that influenced the generation of 9,10-DiHOME within the context of exercise stress. 3,4-diHPV is a major bioactive metabolite of monomeric, oligomeric, and polymeric flavan-3-ols. Limited human data indicate that flavan-3-ol metabolites such as 3,4-diHPV can reach target tissues such as the skin and influence LOX and CYP enzyme systems to decrease proinflammatory oxylipin production (49).

Each of the gut-derived phenolics that were elevated in the urine of the almond group can be linked to PACs and other (poly)phenols present in almonds such as flavonols, flavanones, and phenolic acids (7). To our knowledge, this is the first study to characterize changes in urine gut-derived phenolics after chronic almond intake. The composite variable (with and without hippuric acid) support a significant and distinct increase in a select number of gut-derived phenolics in response to ingesting 57 g almonds daily for 4 weeks. Other studies have evaluated plasma and urine gut-derived phenolic changes in response to acute almond intake (5, 6, 50–52). Many previous studies have correlated blood and urine phenolic metabolites with positive health outcomes (53, 54).

## Conclusion

This study revealed a novel, contrasting effect of 4-weeks intake of almonds compared to cereal bar intake on post-exercise plasma levels of 9,10-DiHOME (lower) and 12,13-DiHOME (higher). These data imply some effect of almond intake on sEH activity, but underlying mechanisms that influence sEH activity *in vivo* are largely unknown. One study showed that in rats treated with antibiotics to deplete gut bacteria, sEH activity was strongly altered suggesting that a gut bacteria-derived factor(s) may be responsible for the effect (55). Our study indicates that 3,4-diHPV should be considered as one of these gut-derived factors. Much remains to be discovered regarding exercise, nutrition, and lifestyle influences on 9,10-DiHOME and 12,13-DiHOME. Both DiHOMEs are involved with neutrophil and monocyte dysfunction in severe burn injury and are significantly higher in COVID-19 patients compared to healthy controls (40, 56). Inhibition of sEH through pharmaceutical and lifestyle interventions is considered a potential therapeutic strategy to mitigate deleterious outcomes from elevated DiHOMES (39). Despite these linkages between both DiHOMES and trauma and disease states, human studies indicate that 12,13-DiHOME is related to improved mitochondrial function, reduced fat mass, fasting insulin and triglycerides, and is elevated in the blood compartment after an acute bout of exercise in both male and female subjects (57). In general, the elevated post-exercise plasma levels of 12,13-DiHOME with almond intake support positive metabolic outcomes for adults engaging in unaccustomed eccentric exercise bouts. Other almond-related benefits for exercisers revealed in this study include reduced feelings of fatigue and tension, better leg-back strength during recovery, and decreased muscle damage during the first day of recovery.

## Data availability statement

The original contributions presented in this study are included in the article/Supplementary material, further inquiries can be directed to the corresponding author.

## Ethics statement

The studies involving human participants were reviewed and approved by IRB, Appalachian State University. The patients/participants provided their written informed consent to participate in this study.

## Author contributions

DN, QZ, and CK designed the research. DN and CS conducted the research. DN, QZ, CK, CS, AO, AL, and MW analyzed the samples and conducted the data analysis. DN, QZ, CK, CS, AO, and AL wrote and edited the manuscript. DN had primary responsibility for final content. All authors read and approved the final manuscript.

## Supplementary material

Supplementary Data Sheet 1 Identified gut-derived phenolics and reference standards library.

Supplementary Data Sheet 2 Identified oxylipins.
